# Supplementary material for: The Diabetes Remission in India (DiRemI) study: Protocol for a prospective matched-control trial
Source: PLoS One. 2024 Jun 28;19(6):e0306394. doi: 10.1371/journal.pone.0306394 (PMC11213318; doi:10.1371/journal.pone.0306394)
Supplement: S1 File — (PDF) [file pone.0306394.s001.pdf]

FULL DETAILS (Read-only) -> Click Here to Create PDF for Current Dataset of Trial

**CTRI No**	**CTRI/2023/06/053885** [Registered on: 14/06/2023] **Trial Registered Prospectively**
**Acknowledgement Number**	REF/2023/05/067822
**Last Modified On:**	10/07/2023
**Post Graduate Thesis**	No
**Type of Trial**	Interventional
**Type of Study**	Yoga & Naturopathy<br>Behavioral<br>Nutraceutical<br>Other (Specify) [Lifestyle modification]
**Study Design**	Non-randomized, Active Controlled Trial
**Public Title of Study**<br>Clarification(s) with Reply<br>Modification(s)	Effect of Freedom from diabetes protocol on weight loss and HbA1c improvement in patient with Type 2 diabetes in India
**Scientific Title of Study**	Efficacy of lifestyle intervention program on management & remission of type 2 diabetes and its complications – DiRemI study
**Trial Acronym**	DiRemI
**Secondary IDs if Any**	Secondary ID: Nil; Identifier: NIL

Secondary IDs if Any

Secondary ID	Identifier
Nil	NIL

Details of Principal Investigator or overall Trial Coordinator (multi-center study)
Clarification(s) with Reply
Modification(s)

**Name**	Dr Pramod Tripathi
**Designation**	Director
**Affliation**	Freedom From Diabetes Research Foundation
**Address**	Research department, Freedom from Diabetes Research Foundation, First floor, Room no.1,Parth, Ghodke Chowk Prabhat Road, Pune<br><br>Pune<br>MAHARASHTRA<br>411004<br>India
**Phone**	
**Fax**	
**Email**	drpramod@freedomfromdiabetes.org

Details Contact Person Scientific Query
Clarification(s) with Reply
Modification(s)

**Name**	Dr Nidhi Kadam
**Designation**	Research Manager
**Affliation**	Freedom From Diabetes Research Foundation
**Address**	Research department, Freedom from Diabetes Research Foundation, First floor, Room no.7,Parth, Ghodke Chowk Prabhat Road, Pune<br><br>Pune<br>MAHARASHTRA<br>411004<br>India
**Phone**	9822792772
**Fax**	
**Email**	research@freedomfromdiabetes.org

Details Contact Person Public Query
Clarification(s) with Reply
Modification(s)

**Name**	Dr Nidhi Kadam
**Designation**	Research Manager
**Affliation**	Freedom From Diabetes Research Foundation
**Address**	Research department, Freedom from Diabetes Research Foundation, First floor, Room no.7,Parth, Ghodke Chowk Prabhat Road, Pune

	Pune<br>MAHARASHTRA<br>411004<br>India
**Phone**	9822792772
**Fax**	
**Email**	research@freedomfromdiabetes.org

**Source of Monetary or Material Support**	Freedom from diabetes research foundation (Host Institution)
**Primary Sponsor**	**Name**: Freedom from diabetes research foundation<br>**Address**: Parth, Ghodke Chowk, Prabhat Road, Pune<br>**Type of Sponsor**: Research institution
**Details of Secondary Sponsor**	Name: NIL<br>Address: NIL
**Countries of Recruitment**	India

Sites of Study
Clarification(s) with Reply
Modification(s)

No of Sites = **1**

Name of Principal Investigator	Name of Site	Site Address	Phone/Fax/Email
Dr Nidhi Kadam	Freedom from Diabetes Clinic	Research department, Freedom from Diabetes Research Foundation, First floor, Room no.7,Parth, Ghodke Chowk Prabhat Road, Pune<br>Pune<br>MAHARASHTRA	9822792772<br><br>research@freedomfromdiabetes.org

Details of Ethics Committee

No of Ethics Committees= **1**

Name of Committee	Ethics Committee registered with DHR /CDSCO or not	Ethics Committee Registration No.	Approval Status	Date of Approval	Approval Document	Is IEC?
Freedom from Diabetes Research Foundation-Institutional Ethics Committee	Yes	EC/NEW/INST/2022/2821	Approved	20/03/2023	Approval File	No

Regulatory Clearance Status from DCGI

Status	Date	Aproval Document
Not Applicable	No Date Specified	No File Uploaded

Health Condition / Problems Studied

Health Type	Condition
Patients	**(1) ICD-10 Condition:** E116||Type 2 diabetes mellitus with other specified complications,

Intervention / Comparator Agent
Clarification(s) with Reply
Modification(s)

Type	Name	Details
Intervention	Lifestyle modification	The duration of the intervention is 1-year. Intervention will consist of Plant-based diet, Physical activity (strength, stamina flexibility exercises including yoga), stress management (Counselling sessions and meditation), routine medical management
Comparator Agent	Routine medical Care	The duration of the intervention is 1-year. Control group will receive Routine medical care (RMC) aiming at education, support, and medical management of diabetes and associated co-morbidities, based on current clinical guidelines

Inclusion Criteria
Clarification(s) with Reply
Modification(s)

**Age From**	30.00 Year(s)
**Age To**	70.00 Year(s)
**Gender**	Both
**Details**	1. Written informed consent.<br>2. Men and women aged 30-70 years.<br>3. T2D duration <15 years.<br>4. Confirmed diagnosis of type 2 Diabetes- All patients on treatment with oral hypoglycemic agents and/or Insulin or any Ayurvedic anti-diabetic medications or HbA1c ≥ 48 mmol/mol (6.5%) with or without medication.<br>5. Body mass index (BMI) >25 kg/m2 and <35 kg/m2.<br>6. Agree to examinations as per the study protocol, undergo the lifestyle intervention program & post-program evaluation.<br>7. Well-oriented in time, space & as a person.

Exclusion Criteria

**Details**	1. Type 1 diabetes (due to autoimmune b-cell destruction, usually leading to absolute insulin deficiency, including latent autoimmune diabetes of adulthood).<br>2. Specific types of diabetes due to other causes, e.g., monogenic diabetes syndromes (such as neonatal diabetes and maturity-onset diabetes of the young), diseases of the exocrine pancreas (such as cystic fibrosis and pancreatitis), and drug- or chemical-induced diabetes (such as with glucocorticoid use, in the treatment of HIV/AIDS, or after organ transplantation).<br>3. Gestational diabetes mellitus (diabetes diagnosed in the second or third trimester of pregnancy that was not clearly overt diabetes prior to gestation).<br>4. eGFR<45mL/min/1.73m2 and/or albuminuria ≥300mg).<br>5. Known history of retinopathy or neuropathy.<br>6. Previous history of heart attack, cardiac arrhythmia, bypass or stent placement surgery, ischemic heart disease, Angina (Class III & IV), myocardial infarction within the previous 6 months, reported heart failure (reduced LV ejection fraction ≤40%), ECG not within normal limits, reported positive stress test which is moderate to severe.<br>7. A known diagnosis of cancer.<br>8. Pregnant or lactating women.<br>9. Patients who have required hospitalization for any diabetes-related complications in the last 6 months or for depression or are on antipsychotic drugs.<br>10. Physical ailments (like spine issues, arthritis, etc.) that limit the implementation of the exercise protocol.

**Method of Generating Random Sequence**	Computer generated randomization
**Method of Concealment**	An Open list of random numbers
**Blinding/Masking**	Open Label

Primary Outcome

Outcome	TimePoints
At least 10% reduction in weight at one year & HbA1c less than 6.5% or 48 mmol/mol without the use of pharmacotherapy at 1 year.	HbA1c & weight will be measured at baseline, at 6 months & at the end of 12 months

Secondary Outcome

Outcome	TimePoints
a) Mental Health (in terms of reduction in scores below 10 for both anxiety & depression), b) Improvement in Co-morbidities & diabetes-related complications (nephropathy), c) Biochemical parameters e.g.,	For Mental health- At baseline & at 6 months Co-morbidities &

	Improvement in-Serum Lipids, Fasting and post prandial plasma glucose, creatinine/eGFR.	complications- At baseline, 6 months & at 12 months<br>For biochemical parameters- AT baseline, at 6 months and at end of 12 months

**Target Sample Size**	**Total Sample Size=**"360"<br>**Sample Size from India=**"360"<br>**Final Enrollment numbers achieved (Total)=** "Applicable only for Completed/Terminated trials"<br>**Final Enrollment numbers achieved (India)=**"Applicable only for Completed/Terminated trials"
**Phase of Trial**	N/A
**Date of First Enrollment (India)**	01/07/2023
**Date of Study Completion (India)**	Applicable only for Completed/Terminated trials
**Date of First Enrollment (Global)**	If country of recruitment is only India, global date would be not applicable.
**Date of Study Completion (Global)**	Applicable only for Completed/Terminated trials
**Estimated Duration of Trial**	**Years=**"1"<br>**Months=**"6"<br>**Days=**"0"
**Recruitment Status of Trial (Global)**<br>Modification(s)	If country of recruitment is only India, global status would be not applicable.
**Recruitment Status of Trial (India)**	Open to Recruitment
**Publication Details**<br>Clarification(s) with Reply<br>Modification(s)	Not yet
**Individual Participant Data (IPD) Sharing Statement**	**Will individual participant data (IPD) be shared publicly (including data dictionaries)?**<br><br>**Response** - NO
**Result Disclosure**	**Do you wish to upload results?**<br><br>**Response** - Summary results have not yet been disclosed
**Brief Summary**	The proposed study is a open labelled, non-randomized, parallel-controlled trial that aims to study the effectiveness of DiRemI protocol in improving glycemic control in patients with Type 2 diabetes. The primary hypothesis is that the intervention which comprises of lifestyle modification with a plant-based diet, exercise, psychological support, and medical management, will result in weight-loss induced diabetes remission and improvement in glycemic control of patient with type 2 diabetes. The primary objective is to implement the intervention that will aim for the remission of diabetes by achieving reduction in weight at one year and HbA1c < 6.5% or 48 mmol/mol without the use of pharmacotherapy at 1 year. The secondary objective is to assess the effect of the intervention on other aspects of diabetes outcomes such as- a) Mental Health (in terms of anxiety and depression), b) Co-morbidities and diabetes-related complications (nephropathy), c) Biochemical parameters e.g., Serum Lipids, Fasting and post prandial plasma glucose, creatinine/eGFR. The duration of the intervention will be 18 months (12 months intervention + 6 month follow-up). Data will be collected at 3 time points: at the baseline, at 6-months, and at the end of 1 year of intervention. Adherence data will be collected from patients.
