# Supplementary material for: The Diabetes Remission in India (DiRemI) study: Protocol for a prospective matched-control trial
Source: PLoS One. 2024 Jun 28;19(6):e0306394. doi: 10.1371/journal.pone.0306394 (PMC11213318; doi:10.1371/journal.pone.0306394)
Supplement: S1 Appendix — (PDF) [file pone.0306394.s003.pdf]

Detail instructions to the participants for accurate measurements and reporting of the weight (kgs)

Getting Ready

1. Before using the scale for the first time, check the battery compartment and remove any plastic insulation strips or packaging that may be in place to prevent battery drainage during shipping/storage.
2. Place the scale on a hard surface. Do not place it on a carpeted floor.
3. Get ready to measure your weight. Remove your shoes, and any heavy clothing like a jacket or sweatshirt, and empty your pockets.
4. Turn on the scale and make sure it is set to record in kilograms (kg):
   - Press the UNIT key on the back of the scale to change the scale's displayed unit of measure.
   - If the scale is turned off then back on, it will default to the last unit used.

Measuring Your Weight

I. First Measurement:

   a. Step on the scale. Try to stand in the center of the scale and make sure you are not touching or holding anything (e.g. a wall, chair). Take note of the number.
   b. Step off and record the measurement below (Weight 1).
   c. Make sure the scale is back to 0 before stepping on for your second measurement.

II. Second Measurement:

   a. Step on the scale. Try to stand in the center of the scale and make sure you are not touching or holding anything (e.g. a wall, chair). Take note of the number.
   b. Step off and record the measurement below (Weight 2).

III. Third Measurement (only needed if first and second measurements are more than 0.2 kg different)

   a. Make sure the scale is back to 0 before stepping up for your third measurement.
   b. Step on the scale. Try to stand in the center of the scale and make sure you are not touching or holding anything (e.g. a wall, chair). Take note of the number.
   c. Step off and record the measurement below (Weight 3).

Date of Measurement:

Weight 1: …….kg

Weight 2: …….kg

Weight 3: …….kg
