# Supplementary material for: The Diabetes Remission in India (DiRemI) study: Protocol for a prospective matched-control trial
Source: PLoS One. 2024 Jun 28;19(6):e0306394. doi: 10.1371/journal.pone.0306394 (PMC11213318; doi:10.1371/journal.pone.0306394)
Supplement: S2 Appendix — (PDF) [file pone.0306394.s004.pdf]

Your Diet Plan

3 Meal Plan (BMI>25 kg/m^2)

Name: XXX **Start Date: XXX**

6:00 - 7:30 AM	PRE-BREAKFAST
	**Smoothie**<br>Green smoothie **500 ml**<br>**If green leafy vegetables are not available go for WHITE SMOOTHIE**
**8:00 - 9:00 AM**	**BREAKFAST**
**25-25-50%**<br>**Formula**	**Salads**<br>Salad **1 Bowl (Katori)**
**[25% Salads +**<br>**25% Sprouts +**<br>**50% Dal Based Items (Cooked)]**	**Sprouts**<br>Raw Sprouts **1 Bowl (Katori)**<br>**Dal Based Items (Cooked) 1 Portion**

1:00 - 2:00 PM	LUNCH
**25% Formula**<br><br>**[25% Salads + 25% Vegetables (Cooked) + 25% Dal Based Items (Cooked) + 25% Grain Based Items (Cooked)]**	**Salads 1 Bowl**<br><br>**Vegetables (Cooked)**<br>Locally Available Vegetables **1 Bowl**<br><br>**Dal Based Items (Cooked)**<br><br>Standard Cooked Dals **1 Portion**<br><br>**Grain Based Items (Cooked) 1 Portion**<br><br>**If you still feel hungry, repeat one more portion of the above.**
4:00 - 4:30 PM	EVENING
	**Smoothie**<br>Green smoothie **500 ml**<br><br>**Nuts and Seeds**<br><br>Almonds Soaked **4-5 Nos.** + Mixed Seeds (Cucumber, Flax, Gardencress, Hemp, Melon, Pumpkin, Sesame, Sunflower, Watermelon) **4 Teaspoon** +Walnuts halves Soaked **5-6 Nos.**<br><br>**If green leafy vegetables are not available go for WHITE SMOOTHIE**
7:00 - 8:00 PM	DINNER
	**Same as lunch.**

General Instructions :

START	AVOID	TRACK
Take the Smoothie empty stomach in the early morning. Can consume black tea/coffee OR with plant-based milk like coconut and almond or herbal tea 30 min after smoothie	Table sugar, jaggery, honey,candy and sweets, sugary and aerated beverages	Update your diet details & weight at least once a week
Consume filtered/cold pressed oils - groundnut, sesame, mustard, coconut or extra virgin olive oil-3-4 teaspoons/day	High Glycemic Grains (puffed wheat/ rice/maize, cornflakes, instant oats) Fruits (watermelon, overripe banana) and Vegetables (white potato, pumpkin, beetroot, yam, arbi)	Drink approx. 1 glass per 10kg body weight water per day. A little more is fine
Finish dinner before 9 p.m. (preferably between 7- 8.30 p.m.) and maintain your mealtimings	Animal Product-Non-veg & milk (cow - A1, A2/buffalo) and all milk products.	Make sure you complete 7-8 hours of sound sleep. Sleep by 11 p.m. at the latest
Follow the rule of 4th morsel which says the first 3 morsels need to be salad, cooked vegetable & dal in that order. Grain should be taken from the 4th morsel.	Deep fried & refined product items (high fat) - like samosa,vada, chips, (Mixture), bhujia sev bread, biscuits (maida), white rice and refined oils. etc	Eat peacefully. Chew properly till you make it liquid. Eat only one morsel at a time which means only after you have gulped down one morsel then pick up the next one.

Raw & Cooked Portion Sizes with Approximate Macronutrients

Raw Portion size	Cooked Portion size (1 cup =150 ml)	Carbs (g)	Proteins(g)	Fat (g)	Energy (Kcal)
Cereals/Grains (30 g)	6 inch roti or 1 cup	22	3	0.5	100
Beans/Legumes/Pulses (30 g)	1 cup (medium consistency)	17	7	0.5	100
Vegetables (100 g)	1- 1.25 cups (depending on consistency)	5 - 10	2	0.3	20 - 40
Leafy Vegetables (100 g)	1 cup	6	3	0.3	40 - 50
Fruits (100 g)	1 small to medium	15	0	0	50
Nuts (15 g)	10-15 numbers	2	2.5	9	100
Seeds (15 g)	2 tablespoons.	2.5	4.5	6.5	85
Salads (100 g)	--	5 - 10	2	0.3	20 - 40

Your Exercise Plan
(BMI >25)

Day/s	Type	Duration
6 days/week<br>Monday to Saturday	Activation Circulation Exercises and Anti-Gravity Exercises	45-60 min/day

Name of Exercise	Approx. Duration Including Breaks	Repetitions (counts)	Sets (number)	Hold (seconds)
World's Best Warm-up	5-10 min	NA	NA	NA
Supported Super Brain Yoga	2 min	20	1	NA
Chair Sooryanamaskaras	10-20 min	12	2	NA
Wall Push Ups	3 min	10	2	NA
Staircase Climbing (Without Weighted Vest)	5-10 min	50-100	1	NA
Nitric Oxide Dump	7-8 min	10	2	NA
Walking (With Weighted Vest)	20 min	NA	NA	NA

General Instructions -

- If starting exercises after a long time, do them on alternate days to avoid getting excess pain
- Take rest if in pain or experiencing a lot of fatigue. If the pain doesn't go away in 2 days,contact me
- Know your exercise capacity. Adapt and progress gradually. Don't be in a hurry. Don'tpush your body mentally beyond safe levels
- Start with a long version of World's Best Warm Up for the first 2 weeks. Move to a short version for the next 2 weeks
- Do either Staircase Climbing OR Nitric Oxide Dump 1 hour 45 minutes from the start of all meals
- Limit Staircase Climbing to once a day

Your Exercise Plan

Fat Loss

Summary Table -

No. of Day/s	Day/s	Type
3	Monday, Wednesday, Friday	Yoga Asanas
2	Tuesday, Thursday	Strength Exercises
1	Saturday	Stamina Exercises

Day/s	Type	Duration
Monday, Wednesday, Friday	Yoga Asanas	45 min

Day/s	Type	Duration
Tuesday, Thursday	Strength Exercises	15-20 min

Remarks -

- **Use medium to heavy resistance band or light weight Dumbbells of 3 to 5 kg as per your comfort**

Day/s	Type	Duration
Saturday	Stamina Exercises	50-60 min

General Instructions –

- In case of any pain, discomfort, or doubts, get in touch with an expert
- While on 1 meal / Juice Fasting (JF) only the World's Best Warm Up, breathing exercises and Yoga Asanas are recommended in the morning. On JF day, 15-20 min of Dry Rubbing is recommended in the evening

Your Exercise Plan

Muscle Gain

Summary Table -

No. of Day/s	Day/s	Type
3	Monday, Wednesday, Saturday	Strength Exercises
2	Tuesday, Thursday	Yoga Asanas
1	Friday	Stamina Exercises

Week	Day/s	Type	Duration
1	Monday	Strength Exercises – Upper Body	30-40 min
1	Wednesday	Strength Exercises-Core	30-40 min
1	Saturday	Strength Exercises – Lower Body	30-40 min
1	Tuesday, Thursday	Yoga Asanas	15-20 min
1	Friday	Stamina (Cardio) Exercises	30-40 min
2	Monday	Strength Exercises – Upper Body	30-40 min
2	Wednesday	Strength Exercises-Core	30-40 min
2	Saturday	Strength Exercises – Lower Body	30-35 min
2	Tuesday, Thursday	Yoga Asanas	25-30 min
2	Friday	Stamina (Cardio) Exercises	30-35 min
3	Monday	Strength Exercises – Upper Body	25-30 min
3	Wednesday	Strength Exercises-Core	30-40 min
3	Saturday	Strength Exercises – Lower Body	30-40 min
3	Tuesday, Thursday	Yoga Asanas	25-30 min

3	Friday	Stamina (Cardio) Exercises	30-40 min
4	Monday	Strength Exercises – Upper Body	25-30 min
4	Wednesday	Strength Exercises -Core	30-40 min
4	Saturday	Strength Exercises – Lower Body	30-40 min
4	Tuesday, Thursday	Yoga Asanas	30-40 min
4	Friday	Stamina (Cardio) Exercises	30-40 min

General Instructions -

- In case of any pain, discomfort, or doubts, please get in touch with an expert.
- While on 1 meal / Juice Fasting (JF) only the World's Best Warm, breathing exercises and Yoga Asanas are recommended in the morning. On JF day, 15-20 min of Dry Rubbing is recommended in the evening.
